# Plasma ddPCR for etiological diagnosis of focal bacterial infections

**DOI:** 10.3389/fmed.2025.1613077

**Published:** 2025-07-24

**Authors:** Yongfeng Zhang, ShanShan Weng, Xueqing Ma, Ling Lin, Yueliang Chen, Binbin Feng, Jiang Xia, Jiayuan Ye, Hua Zhou, Yunsong Yu, Dongdong Zhao

**Affiliations:** ^1^Department of Infectious Diseases, Jiangshan People’s Hospital, Quzhou, China; ^2^Department of Infectious Diseases, Sir Run Run Shaw Hospital, Zhejiang University School of Medicine, Hangzhou, China; ^3^Key Laboratory of Microbial Technology and Bioinformatics of Zhejiang Province, Hangzhou, China; ^4^Regional Medical Center for National Institute of Respiratory Diseases, Sir Run Run Shaw Hospital, Zhejiang University School of Medicine, Hangzhou, China; ^5^Taizhou Hospital, Zhejiang University, Taizhou, China; ^6^Department of Critical Care Medicine, Sir Run Run Shaw Hospital, Zhejiang University School of Medicine, Hangzhou, China; ^7^Pilot Gene Technologies (Hangzhou) Co., Ltd., Hangzhou, China; ^8^Department of Infectious Diseases, Shangyu People’s Hospital of Shaoxing, Shaoxing University, Shaoxing, Zhejiang, China; ^9^Department of Respiratory and Critical Care Medicine, The First Affiliated Hospital, Zhejiang University School of Medicine, Hangzhou, China

**Keywords:** focal infection, droplet digital PCR, molecular diagnosis, blood samples, focal culture

## Abstract

**Background:**

Plasma droplet digital polymerase chain reaction (ddPCR) has been used for pathogen detection and has shown good diagnostic value, but no studies have yet demonstrated its application in focal infections. Herein a pilot study using plasma ddPCR to diagnose focal infection is discussed.

**Methods:**

Eight patients with a diagnosed focal infection who underwent plasma ddPCR for bacterial detection between 2021 and 2022 in Sir Run Run Shaw hospital were enrolled in this retrospective single-center pilot study. Results of ddPCR and focal cultures were compared as well as the turnaround time of two methods and other clinical data.

**Results:**

In 7/8 cases, plasma ddPCR results were consistent with focal culture results. The turnaround time for ddPCR was 2.5 h, significantly lower than the average time for focal cultures of 2.63 days.

**Conclusion:**

This pilot study indicates the promising value of the plasma ddPCR method in the rapid diagnosis of focal infection. If larger studies support the findings here, this method can help improve diagnostic accuracy and guide treatment decisions in suspected focal infections.

## Introduction

A focal infection occurs when an infection is localized in a specific organ or part of an organ such as brain, liver and kidneys. The pathogenic spectrum of focal infection varies from organ to organ, including bacteria, fungi, viruses, parasites, etc. Bacteria as common causative pathogens for most foci are usually detected by culture while this gold standard method reported insufficient sensitivity (1).

The droplet digital polymerase chain reaction (ddPCR) is an improved technique of conventional PCR with higher sensitivity, better accuracy and more stable amplification of rare target DNA sequences (2, 3). This technology achieves absolute quantification by quantifying nucleic acid molecules at the single-molecule level, and thus can accurately measure when testing low-abundance targets. For example, SARS-CoV-2 can be detected at as low as 0.688 or 1.1 cDNA copies (4). It has been proved to be a potential method for the detection of specific bacteria in the study of bloodstream infections (BSI) (5, 6), demonstrating its diagnostic value for infectious diseases.

From our experience of utilizing plasma ddPCR in clinical settings, we found that bacteria concordant with focal cultures could be detected in the blood samples of some patients who were later confirmed to have focal infection. We therefore performed this retrospective study to assess the possibility of using plasma ddPCR for microbial diagnosis of focal infections caused by bacteria.

## Methods

### Study design

This retrospective review was conducted in Sir Run Run Shaw hospital (a tertiary hospital in Zhejiang province, China) from January 2021 to December 2022. For an accurate assessment, we restricted inclusion criteria to exclude the presence of BSI. Patients were enrolled who met the following criteria concurrently: (1) diagnosed with culture-proven focal infection; (2) paired blood culture (BC) and plasma ddPCR conducted synchronously; (3) blood culture-negative. The demographic data and clinical data of the patients were recorded (Tables 1, 2). Due to the pilot nature of the study and the small number of patients enrolled, clinical data were descriptive only.

**TABLE 1:** Plasma ddPCR results and clinical characteristics.

No	Gender	Age	Diagnosis	Radiology	Invasive procedure or surgery	Result of ddPCR (copies/mL)	AMR genes	Sample type for culture	Result of culture	Result of AST
1	M	43	Acute cholangitis	CECT abdomen drainage	Endoscopic nasobiliary	*K. pneumoniae* (1431)	/	Bile	*K. pneumoniae*	carbapenem- susceptible
2	M	29	Infective endocarditis	Transesophageal echocardiography	Thoracoscopy-assisted mitral valve replacement	*S. aureus* (121)	/	Mitral valve vegetation	*S. aureus*	methicillin- susceptible
3	M	76	Acute cholecystitis	CECT abdomen drainage	Percutaneous transhepatic cholangio	*E. faecalis* (113)	/	Bile	*E. faecalis*	vancomycin- susceptible
4	M	60	Liver abscess	CECT abdomen drainage	Percutaneous abscess	*K. pneumoniae* (399)	/	Pus	*K. pneumoniae*	carbapenem- susceptible
5	F	51	Liver abscess	CECT abdomen drainage	Percutaneous abscess	*K. pneumoniae* (752)	/	Pus	*K. pneumoniae*	carbapenem- susceptible
6	M	57	Prostatitis	Prostate MRI	/	*E. coli* (227)	/	Urine	*E. coli*	carbapenem- susceptible
7	F	53	Liver abscess	CT abdomen and abdominal ultrasound	Percutaneous abscess	*K. pneumoniae* (212)	/	Pus	*K. pneumoniae*	carbapenem- susceptible
8	M	49	Renal abscess	CECT abdomen drainage	Percutaneous abscess		/	Pus	*K. pneumoniae*	carbapenem- resistant

AMR, antimicrobial resistance; AST, aspartate aminotransferase; CECT, contrast-enhanced computed tomography; MRI, magnetic resonance imaging; CT, computed tomography.

**TABLE 2:** The laboratory test results and turnaround times.

No	PCT (ng/ml)	hsCRP (mg/L)	WBC (109/L)	Neutrophil percent (%)	Turnaround time of culture (days)	Delayed days of focal sampling	Days of effective antibiotic treatment before blood sampling	Days of invasive procedures or surgery after blood sampling
1	1.15	4.7	20.8	84	3	0	0	0
2	0.27	99.9	5.7	67.8	2	6	2	6
3	0.42	131.6	16.3	85.8	2	1	2	1
4	0.67	199.9	11.1	82.4	3	2	1	2
5	0.77	97.7	17.3	93.7	2	0	4	0
6	0.52	191.2	18.2	88.3	3	0	1	/
7	0.27	49.9	9.9	86.3	3	1	13	1
8	1.5	285	20.3	92.2	3	1	2	−2
Average	0.70	132.49	14.95	85.06	2.63	1.38	3.13	/
95% CI	(0.33, 1.06)	(57.26, 207.71)	(10.41, 19.49)	(78.40, 91.72)	(2.19, 3.06)	(−0.29, 3.04)	(−0.35, 6,60)	/

PCT, procalcitonin; hsCRP, high sensitivity c-reactive protein; WBC, White Blood Cell; CI, confidence intervals.

### Sample collection and plasma DNA extraction

Peripheral venous blood (4 ml) was drawn from each patient into an ethylenediaminetetraacetate (EDTA)-containing tube after two sets of blood cultures (both aerobic and anaerobic bottles, 10 mL per bottle) were taken from the same catheter or venipuncture. Plasma was immediately isolated after centrifugation at 1,600 × *g*, and 22°C for 15 min. Plasma DNA extraction was completed within 1 h from 2 ml of plasma using a Magnetic Serum/Plasma DNA Kit (TIANGEN Biotech, Beijing, China) and the Auto-Pure20B Nucleic Acid Purification System (Hangzhou Allsheng Instruments Company, Hangzhou, China) following the manufacturer’s protocol. DNA was eluted in 50 μl of elution buffer and isolated DNA was quantified with a UV-Vis spectrophotometer (Thermo Fisher, Massachusetts, US). Each sample was measured three times and the average value was taken. The sample concentration ranged from 12 to 22 ng/uL. Regardless of the DNA concentration, 5 uL of the eluted DNA sample was taken for the next step of ddPCR testing for each test.

### Droplet digital PCR

The multiplex ddPCR testing allowed for the detection of eight common bacteria (*Enterococcus faecium*, *Enterococcus faecalis, Staphylococcus aureus, Streptococcus pneumoniae, Klebsiella pneumoniae, Pseudomonas aeruginosa, Acinetobacter baumannii, Escherichia coli*) and three antimicrobial resistance (AMR) genes (*bla*_*KPC*_*, bla*_*NDM*_*, mecA*) in amounts as small as 50 copies/mL (Pilot Gene Technologies. Hangzhou, China). The whole procedure was performed as previously described (6) with minor modifications. The ddPCR test was performed using the Droplet Digital PCR System (Pilot Gene Technology, Hangzhou, China), according to the manufacturer’s instructions. Briefly, 5 μl of extracted DNA sample was added to 10 μl of the ddPCR premix which includes primers, probes, dNTP mixture, Taq polymerase and other necessary components for PCR amplification. The 15 μl mixture was added into a microfluidic chip and loaded into a DG32 droplet generator for droplet generation. Chips were then amplified in a thermal cycler TC1 with the thermal cycling parameters: 95°C for 5 min, followed by 40 cycles of 95°C for 15 s and 60°C for 30 s. Next, chips were transferred to a chip scanner CS5 for fluorescence signal reading and data analysis using GenePMS software (v2.0.01.20011). Synthesized DNA fragment was used as positive control, and DNase- free water or blood samples from three healthy subjects were used as negative controls.

## Results

Ultimately, eight cases with definite diagnosis of focal infection were enrolled in this retrospective pilot study (Tables 1, 2, and Supplementary Figure 1). All patients had fever but no drop in blood pressure when samples were drawn. All blood cultures came back negative even though 7 of them were drawn two sets (both aerobic and anaerobic bottles). Three patients were diagnosed with liver abscess. The other five patients were diagnosed with acute cholangitis, acute cholecystitis, infective endocarditis, prostatitis and renal abscess, respectively, based on radiology and focal culture results. Except for case No. 6, all patients underwent invasive procedures or surgery for treatment and specimen collection. All cultured bacteria from bile, pus, urine and mitral valve vegetation were included in the ddPCR panel, including *K. pneumoniae* (*n* = 5), *E. coli* (*n* = 1), *S. aureus* (*n* = 1) and *E. faecalis* (*n* = 1). The ddPCR test reported concordantly positive results with focal culture in 7 cases while failed to detect the causative bacterium (*K. pneumoniae*) in the remaining 1 case (No. 8). The concentration of bacterial DNA copies detected ranged from 113 to 1431 copies/mL (Table 2).

Antimicrobial resistance genes including *bla*_KPC_, *bla*_NDM_, and *mecA* were not detected, consistent with the results of 7 cases in antimicrobial susceptibility testing (AST). In case No. 8, carbapenem-resistant *K. pneumoniae* strain was detected by culture and AST but missed in ddPCR and no *bla*_*KPC*_ or *bla*_*NDM*_ genes were detected. This negative result may be due to the fact that this case was the only one in which drainage was performed 2 days prior to blood collection (Table 2).

Procalcitonin, high-sensitivity C-reactive protein (hs-CRP) as well as blood routine tests were performed in all patients at the time of BC and ddPCR. PCT and hs-CRP were elevated to varying degrees in all patients. The PCT levels of all 8 patients were lower than 2 ng/mL at the time of sampling, suggesting that they were at a moderate risk of sepsis. Among them, the PCT levels of patients No. 2, 3, and 7 were lower than 0.5 ng/mL, indicating a state of localized infection rather than systemic bacterial infection (7). White blood cell counts and neutrophil percentages were elevated in all patients except patient No. 2 who was diagnosed with infective endocarditis.

The turnaround time of focal culture ranged from 2 to 3 days regardless of sample type, with an average time of 2.63 ± 0.62 days, which was significantly higher than that 2.5 h per sample of ddPCR test (Mann-Whitney U test, *P* < 0.0001). All focal samples were obtained no earlier than blood samples, with a maximum interval of 6 days later (No. 2). Late sampling of focal specimens, combined with longer turnaround times of culture, resulted in focal culture results being available an average of 3 days later than ddPCR results.

Prior to the blood draw, all patients received effective antibiotic treatment except patient No. 1. Case No. 8 with negative ddPCR result received effective treatment for 2 days, while 4 of the positive cases exceeded this time, the longest of which even lasted 13 days (No. 7). This suggested that the duration of effective treatment may not affect the results of ddPCR in our study.

## Discussion

Focal infections can cause intermittent bacteremia, which is difficult to detect by blood cultures (8, 9). In this case, however, ddPCR may have an increased capability of pathogen detection because of its ability to identify bacterial DNA, which might have persisted in a patient’s bloodstream after a period of intermittent bacteremia (10). In this pilot study, we reviewed in detail 8 patients with definite diagnosis of focal infection, in an effort to evaluate the robustness of plasma ddPCR results, in the context of a concurrent negative BC. In 7 out of 8 (87.5%) cases we included, causative DNA fragments were detected in peripheral blood samples, i.e., plasma ddPCR results were consistent with focal culture results. This result suggested that it may be a common phenomenon that ddPCR can detect causative bacteria of focal infection in peripheral blood.

This approach, if successfully applied, would bring many advantages in clinical practice. Traditionally, the diagnosis of focal infection has relied on radiology examination and focal cultures. However, when focal infection is in its early stage, radiological methods may fail to detect the foci due to the lack of substantial anatomical changes (11), making localization and sampling of the infection difficult. Furthermore, sampling of deep site infection is often difficult and traumatic. When surgery is required for sampling, it might be delayed according to clinical condition. As in our study, focal sampling was no earlier than blood collections in all cases and it took 6 days in case No. 2 after the blood draw to get vegetations for culture by mitral valve surgery. Plasma ddPCR, however, could be performed rapidly and less invasively. Compared to culture, ddPCR method could reduce turnaround time from days to hours. Timely sampling along with a short turnaround time enables this method to achieve rapid etiological diagnosis and thus antibiotic de-escalation or treatment adjustment.

Our novel findings provide the first step evidence that plasma ddPCR has the potential to act like liquid biopsy for diagnosing and monitoring remote foci in a non-invasive manner as shown with the Karius test in pneumonia, bone and joint infections and central nervous system infections (12–14). However, there were still many issues to be further explored. First, due to the limitation of retrospective design, this study had potential selection bias and reporting bias. Second, due to the small sample size of our study, we were not yet able to evaluate the efficacy of plasma ddPCR in the diagnosis of focal infection as well as to identify factors that affecting ddPCR results. Previous study (6) has reported a presumptive false positive rate of 7.5% using similar PCR panels for the diagnosis of BSI. The possible reasons of false-positive results might be explained by the presence of possible contamination or DNAemia which presents circulating cell-free DNA from dead bacteria in the absence of infection (15). Similar cases may arise when the application of plasma ddPCR is expanded to focal infection. ddPCR testing of focal fluid or pus samples can be used to verify focal culture-negative cases. In addition, using two or more sets of primers and probes to detect the same pathogen by plasma ddPCR may reduce the false positive rate, but this idea also needs to be verified by further research.

In conclusion, our study demonstrates the etiological diagnostic value of plasma ddPCR in different types of focal infections. This method is not only highly sensitive, but also less invasive and has a shorter turnaround time than sample collection from infected organs, so its early application may help improve patient outcomes and reduce healthcare burden. To promote this method in the clinic, further investigations with larger sample sizes are required to evaluate the diagnostic efficiency, clinical efficacy, cost-effectiveness, and optimal timing of the assay.

## Conclusion

The ultra-high sensitivity of plasma ddPCR detection technology for cell-free DNA in peripheral blood samples provides the possibility of etiological diagnosis of even focal infection. If studies with larger sample size support the findings here, it will be of great help to judge whether patients are infected with common pathogens in clinical practice.

## Data Availability

The raw data supporting the conclusions of this article will be made available by the authors, without undue reservation.

## Supplementary material

Supplementary Figure 1 Timeline diagram.
