# Supplementary figures and images for: Plasma ddPCR for etiological diagnosis of focal bacterial infections
Source: Front Med (Lausanne). 2025 Jul 24;12:1613077. doi: 10.3389/fmed.2025.1613077 (PMC12328317; doi:10.3389/fmed.2025.1613077)

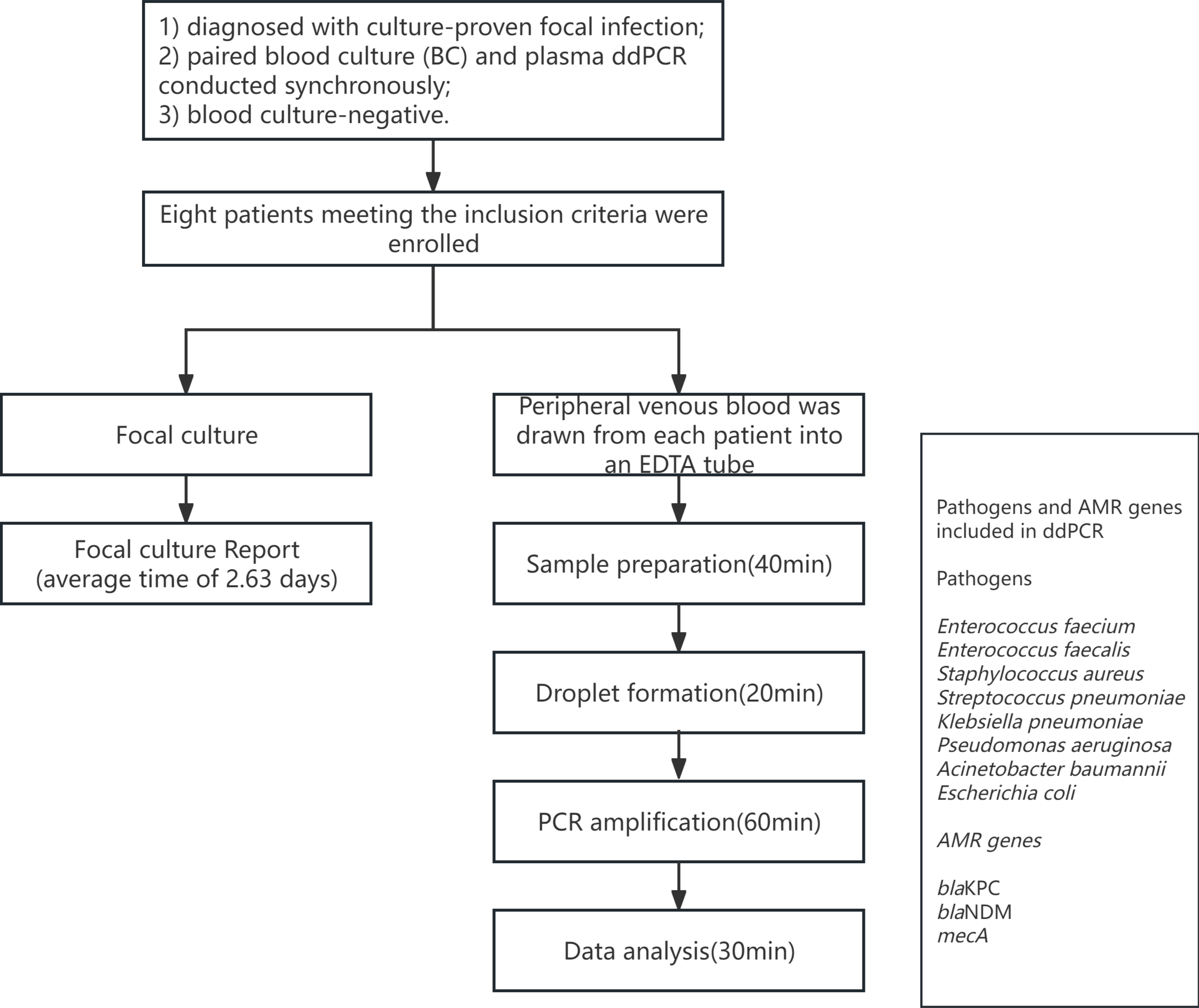

Supplement: Supplementary Figure 1 — Timeline diagram. [file Data_Sheet_1.pdf]
